# Five-year trend of acquired antitubercular drug resistance in patients attending a tertiary care hospital at Dehradun (Uttarakhand)

**DOI:** 10.4103/0970-2113.56342

**Published:** 2009

**Authors:** Jagdish Rawat, G. Sindhwani, Ruchi Juyal, Ruchi Dua

**Affiliations:** *Department of Pulmonary Medicine, Himalayan Institute of Medical Sciences, Swami Rama Nagar, Doiwala, Dehradun (Uttaranchal), India*; 1 *Department of Community Medicine, Himalayan Institute of Medical Sciences, Swami Rama Nagar, Doiwala, Dehradun (Uttaranchal), India*

**Keywords:** Acquired drug resistance, multi-drug resistance, tuberculosis

## Abstract

**Background/Aim:**

To study the prevalence and trend of acquired drug resistance to the first line antitubercular drugs.

**Materials and Methods:**

Sputum of 215 previously treated adult pulmonary tuberculosis (TB) patients over a period of 2002-2006 were subjected to culture and sensitivity testing against common antitubercular drugs.

**Result:**

Growth of *Mycobacterium tuberculosis* was obtained from sputum specimen of 184 (85.58%) of the 215 patients who were studied; Overall, 113 (62.77%) of these were resistant to at least one antitubercular drug. Resistance to isoniazid was most common (62.22%) followed by rifampicin (57.22%). Multidrug resistance (MDR) was observed in 103 (57.22%) cases. During the five-year study period, an increasing trend in drug resistance including MDR-TB was observed.

**Conclusion:**

This study showed increasing trend in drug resistance including MDR-TB in five years.

## INTRODUCTION

Reducing the global burden of tuberculosis (TB) remains a paramount public health priority. Recent estimates are that 32% of the world population, (1.86 billion) is infected with *Mycobacterium tuberculosis* and 8.4 million new cases were observed in 2000.[1] India accounts for nearly 30% of the global TB burden. Drug-resistant tuberculosis has been reported since the early days of introduction of anti-tubercular chemotherapy. But, recently, multidrug-resistant (MDR)-TB has been an area of growing concern, and is posing a threat to the global efforts of TB control.

Several studies[2–6] conducted by various authors in different parts of country reveal the total prevalence of primary/initial MDR-TB as 3% (0-5%) and the rate of acquired MDR-TB in India varies from 6% to 100%. In a survey done by WHO-IUATLD in India, the median prevalence of primary and acquired MDR-TB was found to be 3%-4% and 25%, respectively.[7]

The response of patients with MDR-TB to treatment is poor and the mortality rate is usually high. Because these patients need to be treated with expensive and more toxic second-line drugs and may require hospitalization to manage their toxic reactions and other complications; they require a sizeable proportion of health care resources.

Furthermore, an alarming increase in infection due to the human immunodeficiency virus (HIV) has accelerated this situation. There is a grave concern in India regarding the increase in HIV-associated TB and the emergence of MDR-TB both in magnitude and severity.

As no information on acquired drug resistance (ADR) was available from Uttarakhand, a retrospective survey of antitubercular drug resistance was conducted for a period from 2002 to 2006, with the aims to determine the prevalence of re-treatment drug resistance rate of *M. tuberculosis* as well as to study the trend of ADR within this period.

## MATERIALS AND METHODS

The study was conducted in Himalayan Institute of Medical Sciences (HIMS), a postgraduate institute and a large referral tertiary care center in Uttarakhand from January 2002 to December 2006. Overall, 215 previously treated, smear-positive pulmonary TB patients aged 15 years and above were included in the study. A previously treated patient was defined as a patient treated in the past with one or more course of antitubercular chemotherapy (whether or not treatment had been completed), who is now presenting with symptoms and signs suggestive of TB. The antitubercular drugs were stopped at least one week before subjecting the sputum for culture and sensitivity test. The culture and sensitivity tests were performed on Lowenstein–Jensen medium; these tests were carried out at New Delhi tuberculosis training and demonstration center, Delhi Gate, New Delhi. Positive cultures were subjected to drug susceptibility testing. The culture and sensitivity tests were done against streptomycin (SM), RMP, isoniazid (INH), ethambutol, pyrazinamide and other second-line drugs.

## RESULTS

During the study period, a total of 215 sputum samples were sent for culture and sensitivity. Among 215 patients, 5 were found to be HIV positive and 210 were HIV seronegative. Of these five HIV-positive patients, four specimens were culture positive among which three were susceptible to all the drugs tested, while one strain was found resistant to SM, INH, and RMP. Among 210 HIV-seronegative patients, 180 specimens were culture positive for *M. tuberculosis*. Sixty-seven strains (37.22%) were susceptible to all the drugs tested, and 113 (62.77%) were resistant to at least 1 drug [Table 1]. Resistance to INH was found to be most common (62.22%), followed by RMP (57.22%), SM (22.22%), ethambutol (10%) and pyrazinamide (2.77%). Only 9 cases (5%) revealed resistance to 1 drug, while 104 cases (57.73%) were resistant to 2 or more drugs. MDR-TB was observed in 103 (57.22%) cases [Table 2].

**Table 1:** Prevalence of acquired drug resistance in previously treated pulmonary tuberculosis patients

Pattern of resistance	Study population (n = 180)	%
Fully sensitive	67	37.22
Acquired drug resistance	113	62.77

**Table 2:** Pattern of acquired drug resistance in previously treated pulmonary tuberculosis patients

Pattern of resistance	Study population (n = 180)	% of resistant population (n = 113)
Individual drug resistance	113 (62.77)	-
H	112 (62.22)	112 (99.11)
R	103 (57.22)	103 (91.15)
S	40 (22.22)	40 (35.39)
E	18 (10.00)	18 (15.92)
Z	5 (2.77)	5 (4.42)
Single drug		-
H	9 (5.00)	9 (7.96)
R	0	-
E	0	-
S	0	-
Two drug		-
HR	61 (33.88)	61 (53.98)
HS	0	-
Three drug		-
HRS	25 (13.88)	25 (22.12)
HRE	0	-
SHE	0	-
Four drug		-
HRSE	12 (6.66)	12 (10.61)
MDR	103 (57.22)	103 (91.15)

MDR = Multidrug resistance, H = Isoniazid, R = Rifampicin, E = Ethambutol, S = Streptomycin, Z = Pyrazinamide

Trend in resistance rate among the re-treatment cases from 2002 to 2006 showed a significant increase for any drug as well as for INH + RMP resistance [Table 3].

**Table 3:** Trend in acquired drug resistance to anti-TB drugs in re-treatment cases

Year	No. patients (%)	Any resistance (%)	INH resistance (%)	RMP resistance (%)	R + H resistance (%)
2002	30	17 (56.66)	17 (56.66)	15 (50.00)	15 (50.00)
2003	38	23 (60.52)	23 (60.52)	19 (50.00)	19 (50.00)
2004	35	22 (62.85)	21 (60.72)	18 (52.42)	18 (52.42)
2005	32	21 (65.62)	21 (65.62)	21 (65.62)	21 (65.62)
2006	45	30 (66.66)	30 (66.66)	30 (66.66)	30 (66.66)
Total	180	113	112	103	103

INH = Isoniazid, RMP = Rifampicin

## DISCUSSION

Previous treatment for tuberculosis has been identified as an important risk factor for the acquisition of drug-resistant TB.[8–11] In this study, the overall rate of ADR was 62.77% to one or more antitubercular drugs. Although quite high, the prevalence of ADR observed in this series is comparable, to the rate of 60-85% from different studies[2–5] in India. The high rate of ADR observed in these studies as well as in ours probably reflects the absence of good effective national tuberculosis control program.

Among ADR cases, 57.73% had resistance to 2 or more drugs concomitantly and the most commonly affected drugs were INH and RMP. However, acquired resistance to a single drug alone (5%) was relatively low as compared with data from other parts of the country.[4,5] INH-resistant strains were encountered in 62.22% cases, similar to the observation in other studies from India.[2–5,12–14] The present study has revealed 57.22% acquired resistance to RMP, which is second highest among the levels reported from various centers in our country since 1980.[2,5,15] RMP resistance was always associated with INH resistance in our study. It can therefore be concluded that resistance to RMP is highly predictive of multi-drug resistance in Uttarakhand.

The present study has revealed the increasing trend in drug resistance including MDR-TB during the five-year study period. The longitudinal trend of drug resistance noted by Trivedi and Desai[2] during the 1980s in Gujarat also showed that resistance to RMP increased from 2.8% in 1980 to 37.3% in 1986 and to INH from 34.5% to 55.8%. We have observed no change in SM resistance rate in the present study, which could be due to more frequent use of RMP-containing regimens these days, whereas streptomycin-containing regimens were used frequently before 1980.

We found 5 (2.33%) HIV-positive patients among 215 screened, which confirms the finding of our previous study conducted on patients with TB.[16]

Our study has several limitations. First, we studied only patients with documented positive cultures, not those with negative cultures or those from whom no culture was obtained. Second, nonviable specimens were more likely to be resistant than viable specimen on testing by other methods. Thus, there may have been a slight selection bias against resistant isolates; the actual proportion of patients with resistant isolates may be 2% to 3% higher than the one reported in this investigation. Third, Due to retrospective study, we were unable to separate the patients either they are coming to us from private practitioner or from government sector, so we cannot say rightly by our study that increasing ADR in this region were due to the absence of good, effective national tuberculosis control program in the past.

Detecting increasing drug resistance in TB is important because there are serious consequences of drug resistance, particularly in our setting where the availability of routine susceptibility testing and second-line drugs are limited and the increasing prevalence of HIV might result in rapid dissemination of the problem.

To conclude, the ADR rate of *M. tuberculosis* is quite high in Uttarakhand. Acquired resistance to RMP or in combination with INH (MDR) is also high. This reflects treatment errors that have been made during preceding years. Hence, monitoring the trend of primary drug resistance in Uttarakhand will be critical to determine whether directly observed treatment-short course (DOTS) is able to control the emergence of drug-resistant TB or not.
